# Supplementary material for: Designing 1D correlated-electron states by non-Euclidean topography of 2D monolayers
Source: Nat Commun. 2022 Jun 3;13:3103. doi: 10.1038/s41467-022-30818-2 (PMC9166785; doi:10.1038/s41467-022-30818-2)
Supplement: Supplementary file 1 — Supplementary Information [file 41467_2022_30818_MOESM1_ESM.pdf]

Supplementary Information

Designing 1D correlated-electron states by non-Euclidean topography of 2D monolayers

Sunny Gupta[1], Henry Yu[2], and Boris I. Yakobson[1,3,4*]
[1]*Department of Materials Science and Nanoengineering, Rice University, Houston, TX, 77005 USA*
[2]*Applied Physics Program, Rice University, Houston, TX, 77005 USA*
[3]*Department of Chemistry, Rice University, Houston, TX 77005, USA*
[4]*Smalley-Curl Institute for Nanoscale Science and Technology, Rice University, Houston, TX, 77005 USA*

*Email: biy@rice.edu

Supplementary Note 1 - Continuum model

Supplementary Note 1a - Energy reference for elasticity

The total free energy of a strained 2D material within a region M, neglecting edge elasticity, is expressed as $F = \int_M (\frac{1}{2}\sigma_{ij}u_{ij} + \mu)J dx dy$, with σ_{ij}, u_{ij} the stress and strain tensors respectively, and μ the chemical potential per area of the material at zero strain. The scalar J=det($\partial X_i/\partial x_j$) is the Jacobian determinant relating the curved coordinates (x, y) to the reference coordinates (X, Y)=(x-u_x, y-u_y). The presence of the second term μ signifies the fact that the formation process of the material can affect the strain state of the material. For material grown in highly favorable conditions (μ is very negative), the elastic term $\sigma_{ij}u_{ij}/2$ becomes insignificant and the optimal state corresponds to packing as much material into M, resulting in high compression. On the other hand, for unfavorable growth conditions (μ is very positive), the system is unstable and will dissolve. In this work we highlight the strain engineering of 2D materials, hence, we want to be in a regime wherein the elastic term is prominent. For simplicity we take μ=0, where the material is neither growing nor dissolving, *i.e.* a stable piece of material at rest. The optimal strain state corresponds to “stamping” the material onto the curved substrate and relaxing the lateral displacements.

Supplementary Note 1b - The continuum displacement and strain fields from FvK equations

For a 2D material over a gently varying topography defined as $f(x,y)$, its structural relaxation, on a continuum level, is governed by the Föppl-von Kármán (FvK) equation: [1]
$(\partial_x^2+\partial_y^2)^2\chi+Y(f_{xx}f_{yy}-f_{xy}^2)=0$ (1)
Where subscripts denote partial derivatives. Here $\chi(x, y)$ is the Airy stress function and Y the 2D Young’s modulus. In this work we consider a sinusoidal topography $f(x,y)=h \sin\alpha x \sin\beta y$, where h defines the height, and $\alpha=2\pi/L_x$, $\beta=2\pi/L_y$ defines the lateral periodicity L_x and L_y. Plugging $f(x,y)$ into Supplementary Eq. (1) we will find

$(\partial_x^2+\partial_y^2)^2\chi=(Yh^2\alpha^2\beta^2/2)(\cos 2\alpha x + \cos 2\beta y)$

And, integrating twice, we obtain the Airy function as

$\chi=(Yh^2/32)\,[(\beta/\alpha)^2\cos 2\alpha x + (\alpha/\beta)^2\cos 2\beta y]$ (2)

Integration constants are set to zero to ensure the lowest elastic energy. With the Airy function, the components of the strain tensor can be obtained as $u_{ij}=(1/Y)(\varepsilon_{ik}\varepsilon_{jl} - \nu\delta_{ik}\delta_{jl})\partial_k\partial_l\chi$:

$u_{xx}=(\chi_{yy}-\nu\chi_{xx})/Y=-(h^2/8)(\alpha^2\cos 2\beta y - \nu\beta^2\cos 2\alpha x)$ (3a)

$u_{yy}=(\chi_{xx}-\nu\chi_{yy})/Y=-(h^2/8)(\beta^2\cos 2\alpha x - \nu\alpha^2\cos 2\beta y)$ (3b)

$u_{xy}=0$ (3c)

Supplementary Note 2a - The continuum pseudo-electromagnetic fields from sinusoidal modulation

With the strain fields solved from above, we can easily obtain the pseudo-fields as

$$\varphi_p = -g(u_{ii}/2) = g((1-\nu)/16)h^2(\beta^2\cos 2\alpha x+\alpha^2\cos 2\beta y)$$

$$\mathbf{A}_p = (\beta_0\kappa/a)\,((u_{xx}-u_{yy})/2,\ -u_{xy})$$

$$\rightarrow B_p = (\hbar/e)(\partial_x A_{p,y} - \partial_y A_{p,x}) = -(\beta_0\hbar/\sqrt{2}ae)((1+\nu)/8)h^2\alpha^2\beta \sin 2\alpha y$$

The pseudo-fields derived in main (Eq. (2)) can be obtained by setting $\alpha=\beta$.

Supplementary Note 2b - Anisotropy in pseudo-magnetic field

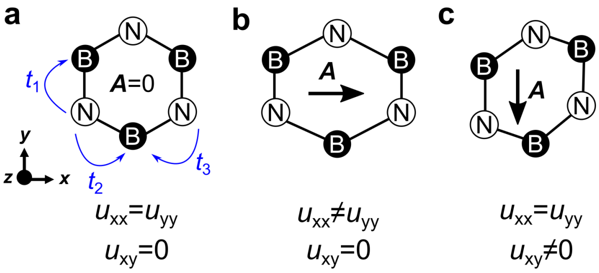

$\boldsymbol{A}$~$[u_{xx}-u_{yy},-u_{xy}]$

$$A_x = \delta t_1 - \frac{1}{2}(\delta t_2 + \delta t_3);\ A_y = \frac{\sqrt{3}}{2}(\delta t_2 - \delta t_3)$$

$$B = \partial_x A_y - \partial_y A_x$$

Supplementary Fig. 1: (a) Isotropic strain ($u_{xx}=u_{yy}, u_{xy}\equiv u_{yx}=0$) leads to a vanishing pseudo-magnetic vector potential $\boldsymbol{A}$. The nearest neighbour hopping terms t_1, t_2, t_3 are also shown. (b), (c) Realizations of the strain tensor u_{ij} that lift the trigonal symmetry and generate $\mathbf{A}$.

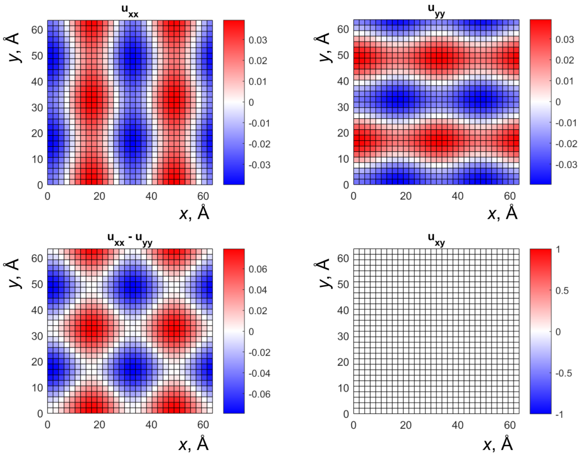

Supplementary Fig. 2: Map of different components of strain tensor, u_{xx}, u_{yy}, u_{xx}-u_{yy} and u_{xy} for geometry in Fig. 1b (main text). Here u_{xy} is equal to zero.

The strain, while symmetric between the *x*, *y* directions, has very different effects on the trigonal lattice and the Hamiltonian of hBN, hence leads to the surprising anisotropic pseudo-magnetic field (PMF) as shown in Fig. 1c (main text). The *x* and *y* directions in fact correspond to the zigzag and armchair directions of the h-BN lattice, respectively, hence are not equivalent. PMF is given by the curl of pseudo-vector potential $\boldsymbol{A}_\mathbf{p}$, which in turn depends on the components of strain tensor *u* as $\boldsymbol{A}_\mathbf{p}$~$[(u_{xx}-u_{yy})/2, -u_{xy}]$. Microscopically, the strain fields generate PMF by changing the nearest neighbor B-N hopping terms *t*, which in pristine BN has a trigonal symmetry (t_1=t_2=t_3) (Supplementary Fig. 1). $\boldsymbol{A}_\mathbf{p}$ in terms of change in hopping terms δ*t* is given by $\boldsymbol{A}_\mathbf{p}$~$[\delta t_1- (\delta t_2+ \delta t_3)/2,\sqrt{3}/2(\delta t_2-\delta t_3)]$. Thus, the strain fields, which generate PMF, break BN's local trigonal symmetry. The interplay between the trigonal symmetry of BN and the symmetry of strain fields creates distinct PMF patterns. One can imagine different strain patterns generating distinct pseudo-vector potential, as shown in Supplementary Fig. 1. In our case, the strain appears symmetric between the *x* and *y* directions, this leads to the shear component of strain tensor u_{xy}=0 (Supplementary Fig. 2) and δt_2=δt_3. Additionally, the present case is analogous to that of Supplementary Fig. 1b, where at most places $u_{xx}\neq u_{yy}$ and strain locally breaks the trigonal symmetry into an effective 2-fold symmetry, such that δt_2=δt_3, while $\delta t_1\neq(\delta t_2+\delta t_3)/2$. This creates non-equivalence in the *x,* and *y-direction*, and thus only $A_{p,x}$ is non-zero. We think that this local anisotropy due to strain fields creates a global anisotropic PMF pattern.

Supplementary Note 2c - Pseudo-magnetic field change due to crystal rotation relative to substrate

The 1D nature of the B_p field (constant along *x*) corresponds to the specific orientation of the substrate. In reality a small rotation *θ* between the substrate and the material will always be present, which can potentially destroy the 1D flat bands. Under a rotation *θ* the PMF is no longer strictly 1D but acquires a finite periodicity. By applying rotation operations on the strain tensors we derived the general B_p field as

$$B_p = (\beta_0\hbar/\sqrt{2}ae)((1+v)/8)h^2 \cdot [\alpha\beta^2 \sin(2\alpha(x\cos\theta+y\sin\theta)) \cdot (3\sin\theta \cos^2\theta - \sin^3\theta) + \alpha^2\beta \sin(2\beta(y\cos\theta-x\sin\theta)) \cdot (3\cos\theta \sin^2\theta - \cos^3\theta)]$$

$$\approx -(\beta_0\hbar/\sqrt{2}ae)((1+v)/8)h^2 \alpha^3 \sin(2\alpha(y-x\theta)), \text{ for small } \theta.$$

Taking $\alpha=\beta$ and $\theta \ll 1$, the second equality shows that the immediate effect of substrate rotation to the PMF pattern is the rotation of patterns and the acquiring a large periodicity $\pi/\alpha\theta$. As shown in Supplementary Fig. 3, the PMF under a small θ = 5° rotation (Supplementary Fig. 3a) still exhibits the 1D-stripe structure, but with periodic modulations. At larger rotations θ = 15° (Supplementary Fig. 3b), the 1D stripes are destroyed, and the PMF exhibits block-like checkerboard pattern. At θ = 30° (Supplementary Fig. 3c) the ZZ line along the 120° direction is now aligned with the bisinusoid square edge and forms strictly 1D stripes in PMF again along the 120° direction. In conclusion the 1D electronic bands should hold under small rotations ($\theta \ll$ 15°) while larger angles ($\theta \approx$ 15°) can destroy it. This pattern repeats every 30° rotation.

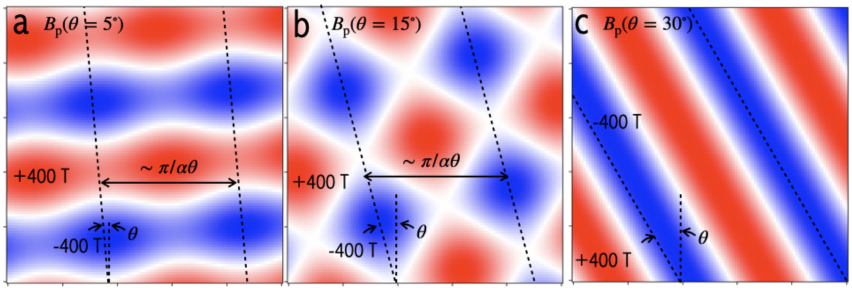

Supplementary Fig. 3: Pseudo-magnetic field due to crystal rotation relative to substrate of (a) $\theta = 5°$, (b) $\theta = 15°$ and (c) $\theta = 30°$.

Effect of hBN-substrate relative rotation on bandwidth: For small rotation θ, the PMF field is slightly perturbed, $B_p \approx B_{p,unpert} + B_{p,pert}$, where $B_{p,unpert} = B_{p,max}\sin(2\alpha y)$, $B_{p,pert} = -2\theta B_{p,max}\alpha x.\cos(2\alpha y)$ and $B_{p,max} = -(\beta_0\hbar/\sqrt{2}ae)((1+v)/8)h^2\alpha^3$. The maximum value of the perturbed B_p field is $B_{p,pert,max} \approx \theta B_{p,max} << B_{p,max}$. For a given length L, the bandwidth $W \propto \exp(-CB_{p,max})$, C is a constant. Adding a small perturbation to $B_{p,max}$ due to small relative rotation of hBN-substrate will change the bandwidth to $W \propto \exp(-CB_{p,max} + C\theta B_{p,max})$. Since, $\theta B_{p,max} << B_{p,max}$ the change in W will be negligible.
For large rotations, $\theta \approx 15^o$, $B_{p,pert,max} \sim B_{p,unpert,max}$, thus W will decrease and become very small, and hopping ratio $t_y/t_x \sim 1$.

Effect of hBN-substrate relative rotation on band gap to remote bands: The band gap to remote bands depends on the periodic confinement distance L along the y-direction in the rotated frame (analogous to that in a particle in a box). Since the substrate rotation does not change L along the y-direction (distance between parallel/isolated blue regions in Supplementary Fig. 3), the band gap to remote bands will not change.

Supplementary Note 2d - Effect of strain imperfections on quasi-1D states

The quasi-1D nature of the eigenstates arises from the anisotropy in the electronic confinement potential due to the pseudo-magnetic field (PMF). Since PMF is only a function of y, the electronic states along the y-direction are like those for a particle in a 1D-finite potential well, and these states are completely localized with no dispersion, which results in hopping $t_y << t_x$. The quasi-1D character will remain, as long as the anisotropic PMF is not significantly perturbed. The effect of 1D-confinement due to PMF can be modelled as a particle in a 1D-finite potential well $-\frac{\hbar^2}{2m}\frac{d^2}{dy^2}\psi(y) + V(y)\psi(y) = E\psi(y)$, where $V(y)$ is the confinement due to PMF. We approximate $V(y)$ as $V(y) = V_{depth}.\exp(-(|x|/\lambda)^k)$, where λ and k are the shape parameter and V_{depth} is the maximum depth of the potential. For the electronic states shown red in Fig. 2a (main text), V_{depth} is 0.5 eV (Fig. 3b) and λ and k are fitted to match the lowest two eigenvalues (E_1 and E_2) with DFTB electronic structure (n = 1, 2 in Fig. 2a). The fitted potential is shown in Supplementary Fig. 4 (a). The quasi-1D nature will remain as long as $E_2 - E_1 > W$ (W is bandwidth) and the full-width at half maximum (FWHM) of the first eigenstate $|\psi_1(y)|^2$ doesn't change significantly. Local strain imperfections will perturb the confinement potential and its effect can then be modelled as perturbations to $V(y)$ (Supplementary Fig. 4 (b),(c)). We find that adding random local perturbation of $0 - \pm V_{depth}$ to $V(y)$ doesn't change the electronic states significantly, $E_2 - E_1 > W$ and the FWHM of $|\psi_1(y)|^2$

(Supplementary Fig. 4(d)) remains the same, signifying that quasi-1D states will remain robust against small local strain imperfections.

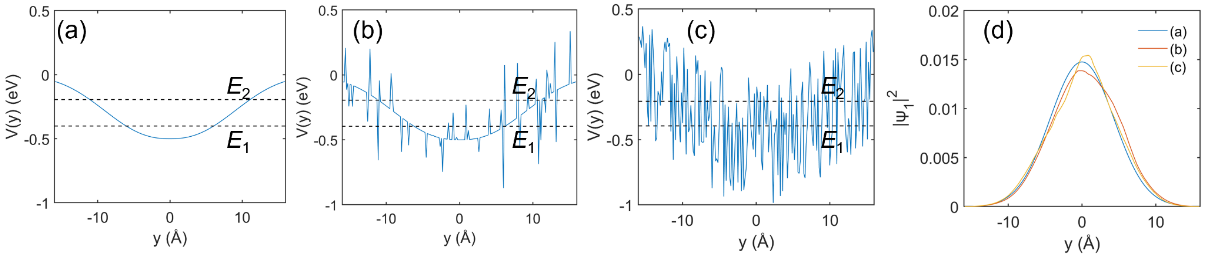

Supplementary Fig. 4: Electronic confinement potential $V(y)$ representing pseudo-magnetic field (PMF), with (a) no perturbation, (b) random localised perturbation, and (c) non-local (broad) perturbation of 0 – ±0.5 eV to $V(y)$. The eigenvalues of the first two lowest eigenstates E_1 and E_2 are also shown. (d) The density profile corresponds to the first eigenstate for potentials in (a)-(c).

Supplementary Note 3a - Building the atomic structure

Relaxing large 2D materials on curved surfaces, at a quantum mechanical level (*e.g.* DFT or DFTB) can be computationally prohibitive. Hence, it is very desirable if we can directly construct a deformed atomic structure from the above strain field. This requires 1) determining the amount of material in the periodic box and 2) find the correct displacement for each atom. From the definition of the strain $u_{ij}=(1/2)(\partial_i u_j + \partial_j u_i + \partial_i f \partial_j f)$ we can integrate the components $u_{xx} = \partial_x u_x+(f_x)^2/2$ and $u_{yy} = \partial_y u_y+(f_y)^2/2$ and find the displacement fields u_x, u_y
$u_x = -(h^2\alpha^2/8)x + (h^2/16\alpha)(\nu\beta^2 - \alpha^2 + \alpha^2\cos 2\beta y)\sin 2\alpha x$ (4a)
$u_y = -(h^2\beta^2/8)y + (h^2/16\beta)(\nu\alpha^2 - \beta^2 + \beta^2\cos 2\alpha x)\sin 2\beta y$ (4b)
Which connects the deformed coordinates (x, y) to the reference coordinates $(X, Y) = (x-u_x, y-u_y)$. The first linear term in Supplementary Eq. (4) determines the overall amount of lateral contraction, and the second term is an oscillating term causing periodic stretching/compression patterns. Hence we can write $(L_x-L_{x0})/L_x=-(h^2\alpha^2/8)$ and $(L_y-L_{y0})/L_y=-(h^2\beta^2/8)$, with L_x and L_y the periodicity of the sinusoid and L_{x0}, L_{y0} the periodicity of the original, reference flake. Plugging in the above definitions $\alpha=2\pi/L_x$, $\beta=2\pi/L_y$ we have (taking $i = x$ or y)
$L_i^2 - L_{i0}L_i + (h^2\pi^2/2) = 0$

$$\rightarrow L_i = [L_{i0} + (L_{i0}^2 - 2\pi^2 h^2)^{1/2}]/2$$

The atomic structure can therefore be constructed as the following:

1) Create a hBN sample with dimensions L_{x0}, L_{y0}
2) Choose desired height h for sinusoid
3) Solve for the optimal L_x, L_y, or equivalently $\alpha=2\pi/L_x$, $\beta=2\pi/L_y$ for the sinusoid
4) Displace each atom from $(X, Y, 0) \rightarrow (X+u_x, Y+u_y, f(X+u_x, Y+u_y))$ with u_x, u_y according to Supplementary Eq. (4).

Supplementary Note 3b - Comments on surface adhesion

The continuum model and the generated atomic structures above assumes perfect conformation of the 2D material on the substrate. This requires that the substrate adhesion

to be sufficiently strong to sustain the surface pressure required to impart the strain pattern. For a 2D material conformed to a curved substrate, the surface pressure can be calculated through $P = -\sigma_{ij} f_{ij}$; plugging in AR=0~0.12 for a bi-sinusoid surface, as we did in this work, the maximum pressure is plotted in Supplementary Fig. 5. For an adhesion energy γ the potential predicts a maximal force $P_{max}=\gamma/l_{eff}$; here the effective length l_{eff}=1.8 Å. In Supplementary Fig. 5 values for γ = 1 ~ 100 meV/Å^2 are shown. For SiO_2 *ab initio* calculation gives γ~20 meV/Å^2, corresponding to P_{max}=11 meV/Å^3; this limits the valid AR range to be below 0.1. For structures with larger AR, substrate materials with stronger adhesion ~100 meV/Å$^{2\,2}$, can be chosen.

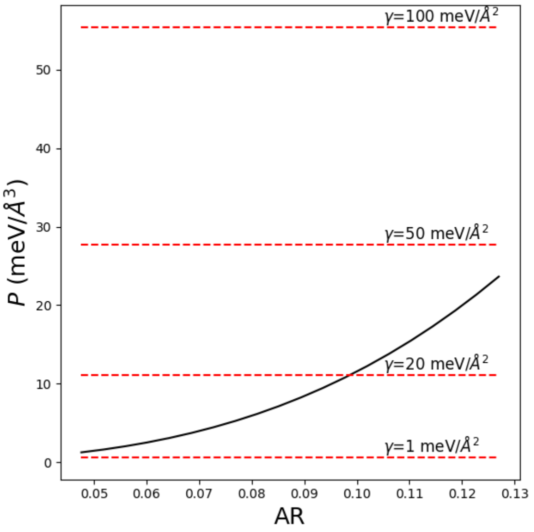

Supplementary Fig. 5: Maximal magnitude of surface pressure (black solid) with respect to aspect ratio (AR) of the sinusoid. Red dashed lines show estimates of vdW force for adhesion energies γ = 1, 20, 50, 100 meV/Å^2.

If the 2D material does not conform to the substrate over a length l, which is just a few lattice constants, and the ratio of l/L <<1, where L is the periodic length modulation, then our analysis and results should still be applicable. If the 2D material, after placing on a patterned substrate, remains suspended in air, and the ratio of l/L~1, then our analysis will not be relevant for such cases. However, recent studies have demonstrated that carefully engineering the substrate can help transition from suspended to fully conformal regimes [3].

Supplementary Note 4 - Methodology: DFTB calculation

DFTB has been successfully applied to study various forms of hBN, for which DFT calculations are intractable. A good agreement between the electronic structure obtained with DFT and DFTB, with a manifold increase in speed, makes DFTB applicable widely and in the present study as well. The electronic structure of the pristine and sinusoidally modulated boron nitride was calculated using the density functional based tight-binding approach implemented in DFTB+[4] using atomic orbital basis. The self consistent charge calculation was performed using a varying k-grid of 6x6x1 - 12x12x1 depending on the size of the unit cell. The maximum angular momentum chosen for B and N atoms was p (l=1). The pairwise B-B, N-N, and B-N Slater koster files (parameterization data) were obtained from the DFTB+ repository, matsci. The electrostatic potential was estimated by taking the

Mulliken-point charges and superposing the corresponding 1/r potentials as implemented in the DFTB+ code. In the code, the 1/r potential is modified to remove the r=0 divergence, and instead plots $1/\sqrt{r^2+\varepsilon^2}$, where $\varepsilon=10^{-4}$.

The integral charge density $\frac{1}{N_k}\sum_{k\in BZ}\sum_{n=1}^{8}|\psi_{nk}(r)|^2$ for the whole flat band shown in red in Fig. 2a (main text) is plotted in Supplementary Fig. 6 , which includes all the 8 states and averaged over 12 k-points in the irreducible Brillouin zone. The charge density is anisotropic and it confirms that all the eigenstates in the flat band are quasi-1D in nature.

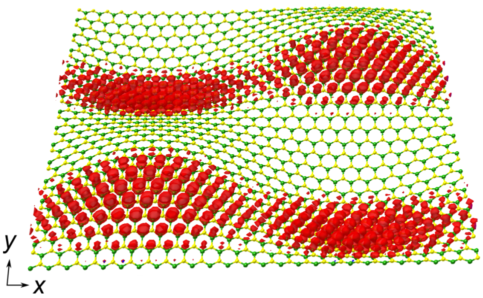

Supplementary Fig. 6: The integral charge density $\frac{1}{N_k}\sum_{k\in BZ}\sum_{n=1}^{8}|\psi_{nk}(r)|^2$ for the 8 states that are part of the flat bands shown in red in Fig. 2a main text.

Supplementary Note 5 - Coarse-grained 8 band tight-binding model

A 8 band tight-binding model was developed to fit the flat bands shown by red color in Fig. 2b (main text). The 8 bands arise from the states localized on the 2 crests and 2 troughs, and each of them being doubly occupied. The crests and troughs act as an artificial “quantum dot”. The TB model can be described by the following Hamiltonian, $H = \Sigma_x t_x c^{\dagger}_{x,y} c_{x+2,y} + \Sigma_y t_y c^{\dagger}_{x,y} c_{x,y+1}$, where t_x and t_y are hopping amplitudes along x-, and y-direction, respectively. The matrix elements of the 8x8 Hamiltonian for the TB model are:

H(1,1) = H(2,2) = H(3,3) = H(4,4) = H(5,5) = H(6,6) = H(7,7) = H(8,8) = 0
H(1,3) = $-t_x$*M*$e^{(ik_x a)}$ - t_x*conj(M)*$e^{(-ik_x a)}$; H(3,1)=conj(H(1,3))
H(2,4) = $-t_x$*M*$e^{(ik_x a)}$ + t_x*conj(M)*$e^{(-ik_x a)}$; H(4,2)=conj(H(2,4))
H(5,7) = $-t_x$*M*$e^{(ik_x a)}$ - t_x*conj(M)*$e^{(-ik_x a)}$; H(7,5)=conj(H(5,7))
H(6,8) = $-t_x$*M*$e^{(ik_x a)}$ + t_x*conj(M)*$e^{(-ik_x a)}$; H(8,6)=conj(H(6,8))
H(1,5) = $-t_y$*$e^{(ik_y b)}$ - t_y*$e^{(-ik_y b)}$; H(5,1)=conj(H(1,5))
H(2,6) = $-t_y$*$e^{(ik_y b)}$ - t_y*$e^{(-ik_y b)}$; H(6,2)=conj(H(2,6))
H(3,7) = $-t_y$*$e^{(ik_y b)}$ - t_y*$e^{(-ik_y b)}$; H(7,3)=conj(H(3,7))
H(4,8) = $-t_y$*$e^{(ik_y b)}$ - t_y*$e^{(-ik_y b)}$; H(8,4)=conj(H(4,8))
M = $e^{(-i\pi/4)}$; $a = L_x/2$; $b = L_y/2$

Supplementary Note 6 - Adhesion energy of SiO_2 on hBN and strain energy of hBN

The 2D material stamped or grown on SiO_2 can delaminate from the substrate, if the desired strain levels are high. We estimated the adhesion energy γ_{ad} of the hBN on SiO_2 and

compared it with the strain energy E_S of hBN.

A 2x2 unit cell of hBN and 7 layers of SiO_2 along [001] was taken to create the hBN|SiO_2 slab geometry. The SiO_2 slab's surface was passivated with hydrogens. The geometry was fully relaxed using first-principles density functional theory (DFT) implemented in VASP[5]. Ion-electron interactions were represented by all-electron projector augmented wave potentials. The generalized gradient approximation (GGA) parameterized by Perdew-Burke-Ernzerhof[6] (PBE) was used to account for the electronic exchange and correlation. A plane wave basis with a kinetic energy cut-off of 500 eV was used for wave functions expansion and a Monkhorst-Pack grid of 12x12x1 *k*-points was used to sample the Brillouin Zone (BZ). A vacuum of 20 Å was used along the direction perpendicular to the slab to reduce the interaction between the periodic images. The structure was relaxed until the Hellmann-Feynman forces on the atoms were less than 0.01 eV/Å. The DFT-D2 method of Grimme was used to include van der Waals interaction.

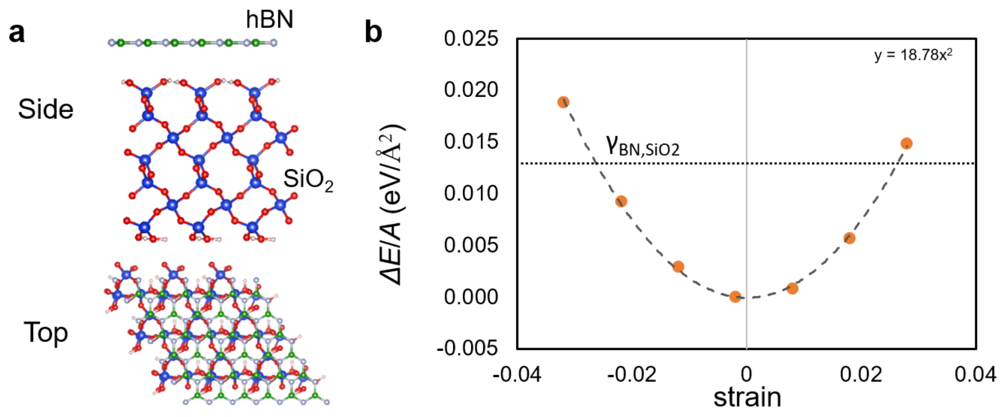

Supplementary Fig. 7: (a) Crystal structure of hBN stacked over SiO_2 slab, where the slab's surface is passivated with hydrogens. (b) Energy of hBN as a function of strain. The dashed line is the fitted curve y=18.78x^2 while the dotted line shows the value of adhesion energy of hBN on SiO_2 $\gamma_{ad.}$

The adhesion energy was estimated using $\gamma_{ad} = (E_{BN} + E_{SiO2}) - E_{slab}$. The strain energy was calculated by evaluating the total energy of the hBN sheet for different biaxially strained structures. The adhesion energy was found to be γ_{ad}~13 meV/Å^2, while the strain energy is E_S~18.92 eV/strain^{2}Å^2.

The flat bands can be accessed by electrostatic doping of hBN. The chemical potential of hBN/SiO_2 system lies within the gap. Our calculation of the position of valence band (VB) and conduction band (CB) of hBN and SiO_2 slab (Supplementary Fig. 8), when referenced to common vacuum shows that hBN|SiO_2 forms a straddled (Type-I) heterostructure, with hBN band extremes straddled between those of SiO_2. To access the strongly correlated physics, hBN must be doped such that chemical potential is at the flat band states, which can be achieved by electrostatic doping, as routinely done for 2D materials, including twisted bilayer TMDs, and graphene.

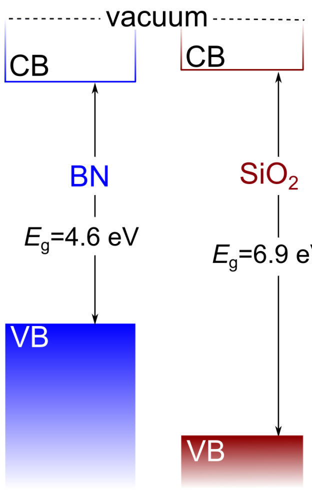

Supplementary Fig. 8: Alignment of valence band (VB) and conduction band (CB) of hBN and SiO_2 slab referenced to common vacuum level.

Supplementary References

1. Landau, L. D., Pitaevskii, L. P., Kosevich, A. M. & Lifshitz, E. M. *Theory of Elasticity: Volume 7*. vol. p. 61, §14 (Butterworth-Heinemann, 1986).
2. Dai, Z., Lu, N., Liechti, K. M. & Huang, R. Mechanics at the interfaces of 2D materials: Challenges and opportunities. *Curr. Opin. Solid State Mater. Sci.* 24, 100837 (2020).
3. Reserbat-Plantey, A. *et al.* Strain Superlattices and Macroscale Suspension of Graphene Induced by Corrugated Substrates. *Nano Lett.* 14, 5044–5051 (2014).
4. Hourahine, B. *et al.* DFTB+, a software package for efficient approximate density functional theory based atomistic simulations. *J. Chem. Phys.* 152, 124101 (2020).
5. Kresse, G. & Furthmüller, J. Efficiency of ab-initio total energy calculations for metals and semiconductors using a plane-wave basis set. *Comput. Mater. Sci.* 6, 15–50 (1996).
6. Perdew, J. P., Burke, K. & Ernzerhof, M. Generalized Gradient Approximation Made Simple. *Phys. Rev. Lett.* 77, 3865–3868 (1996).
